# Supplementary material for: Reconstruction of fish allergenicity from the content and structural traits of the component β-parvalbumin isoforms
Source: Sci Rep. 2019 Nov 8;9:16298. doi: 10.1038/s41598-019-52801-6 (PMC6841720; doi:10.1038/s41598-019-52801-6)

Reconstruction of fish allergenicity from the content and structural traits of the component β-parvalbumin isoforms

Raquel Pérez-Tavarez[1], Mónica Carrera[2], María Pedrosa[3,4], Santiago Quirce[3,4], Rosa Rodriguez-Perez[4] and María Gasset[1]*

[1]Insto Química-Física "Rocasolano", Consejo Superior de Investigaciones Científicas, 28006 Madrid, Spain.
[2]Insto Investigaciones Marinas, Consejo Superior de Investigaciones Científicas, 36208 Vigo, Spain
[3]Dpto de Alergología, Hospital Universitario La Paz, 28046 Madrid, Spain
[4]Insto de Investigación Hospital Universitario La Paz (IdiPaz), 28046 Madrid, Spain

Supplementary Material

Table 1. Clinical data of the patients allergic to fish and their sera features.

Patient	Age (years)	sex	Symptoms after fish ingestion[a]	Ofended fish[b]	Other allergies	SPT[c] (mm) Cod / Tuna	Total IgE (kU/l)	sIgE (kU/l) Cod / Tuna
S1	9.1	M	U, OAS	Hake, tuna	Seafood[d], treenuts	12 / 8	1,020	13.9 / 7.24
S2	10.2	F	U, AE, OAS, V	Hake, megrim	Fruits	5 / 4.5	851	8.42 / 1.68
S3	10.2	M	V	Hake, cod, megrim	Egg, seafood, legumes, treenuts	6 / 0	2,223	8.03 / 2.04
S4	4.6	M	AE, U	Hake	Egg	15 / 12.5	901	15.3 / 1.83
S5	7.8	M	OAS, U	Hake, megrim	Egg, seafood, legumes, treenuts	25.5 / 9.0	278	92.7 / 31.2
S6	10	F	AX	Hake	Seafood	7.5 / 6.5	38.8	5.01 / 1.34
S7	7.2	M	OAS	Hake, megrim	Egg, nuts, legumes	13 / 5.5	1,382	25.7 / 8.72

[a] Abbreviations correspond to: AE: angioedema; OAS: oral allergy syndrome; U: urticaria.

[b] Fish species causing initial symptomatology upon oral exposure.

[c] Mean diameter

[d] Molluscs and crustaceans.

Figure 1S : Original gels of Fig. 2a and 2b.

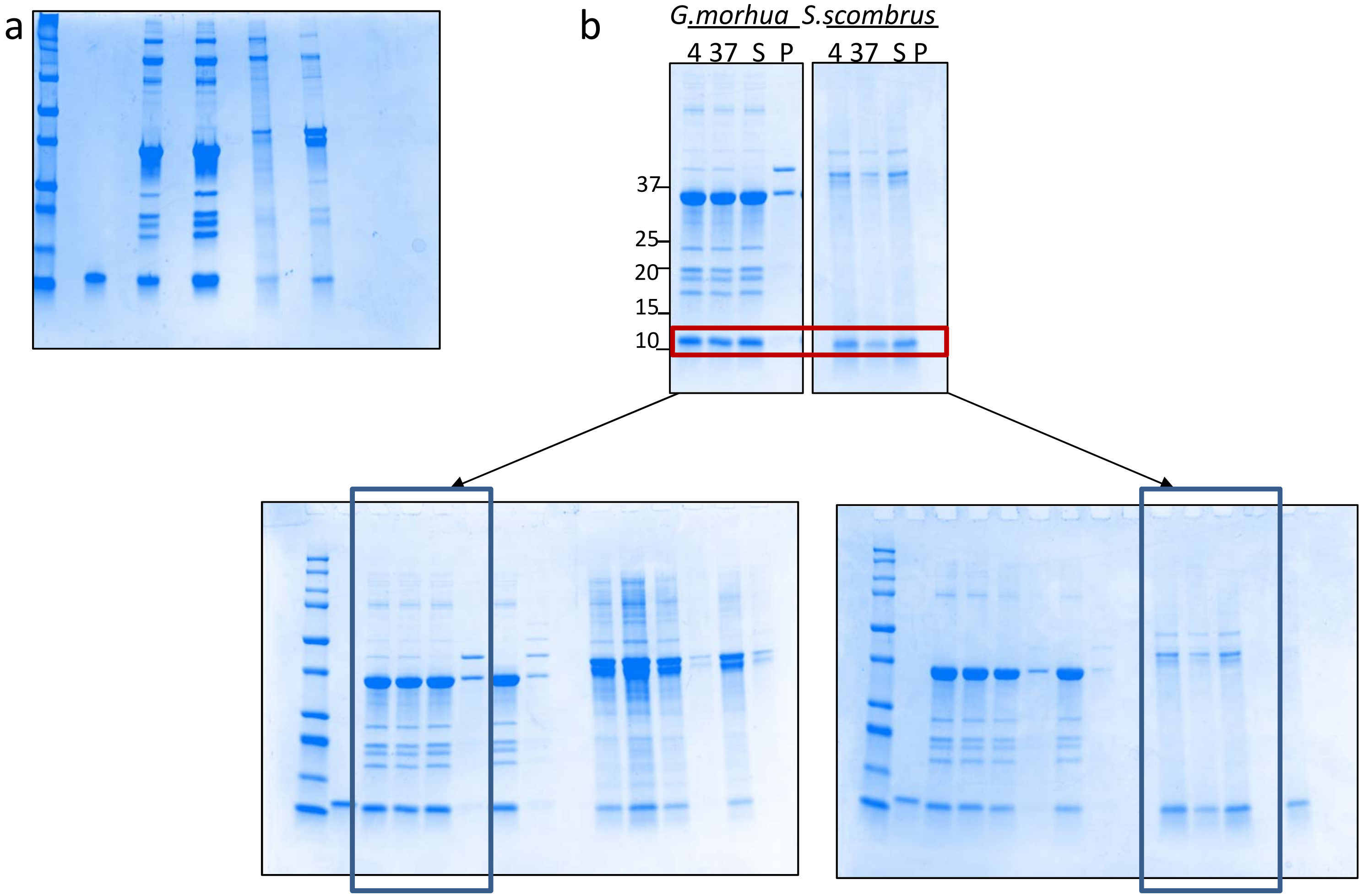

Figure 2S : Original gels and membranes of Fig. 3a and 3b.

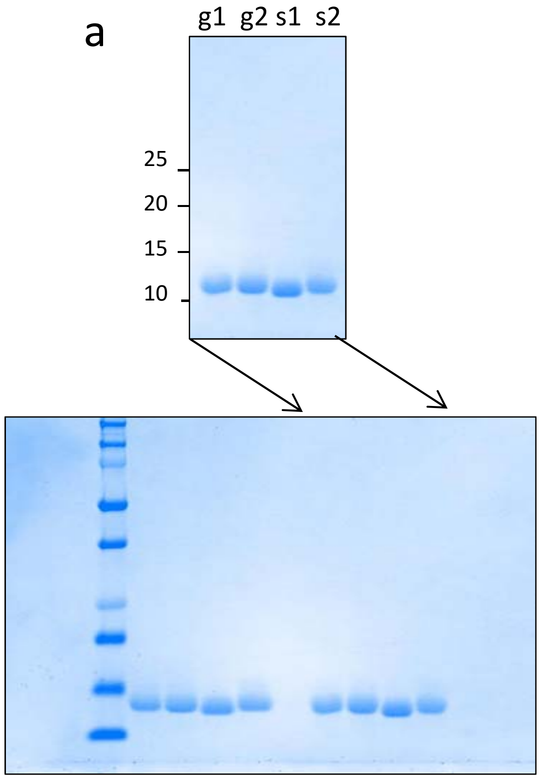

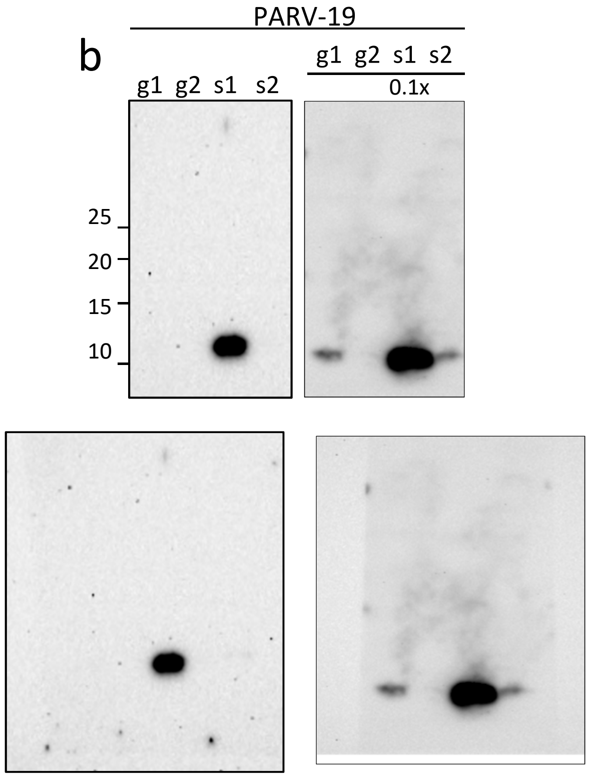

Figure 3S : Original blots used in Fig. 3c.

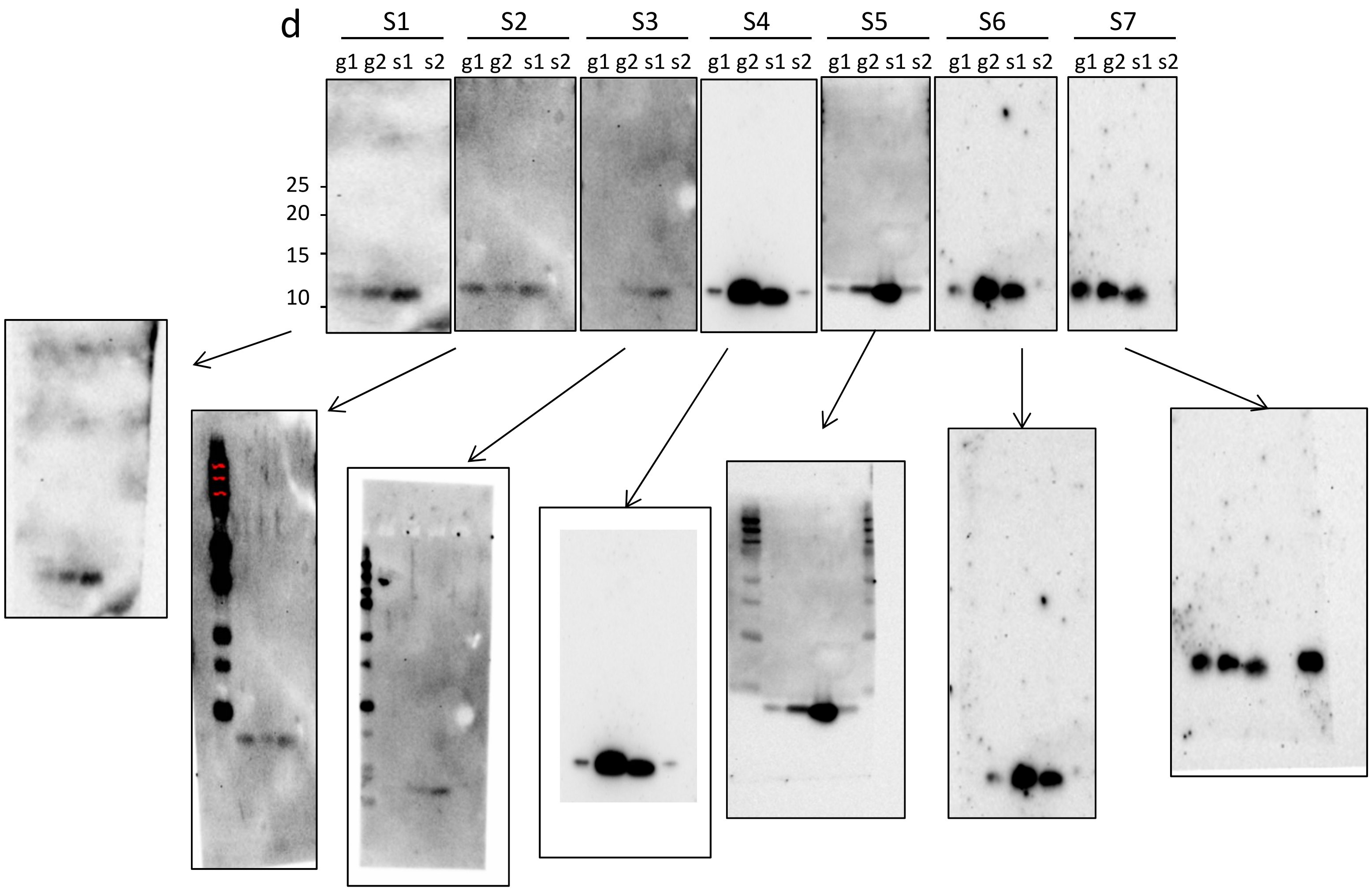

Figure 4S : Full length gel of Fig. 4d.

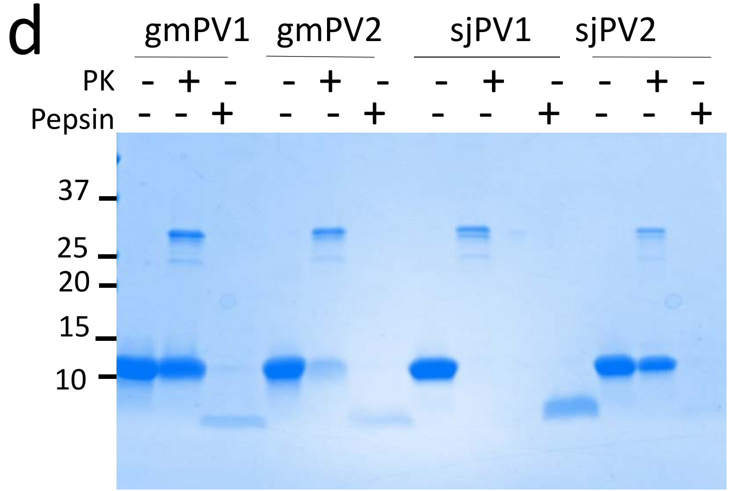

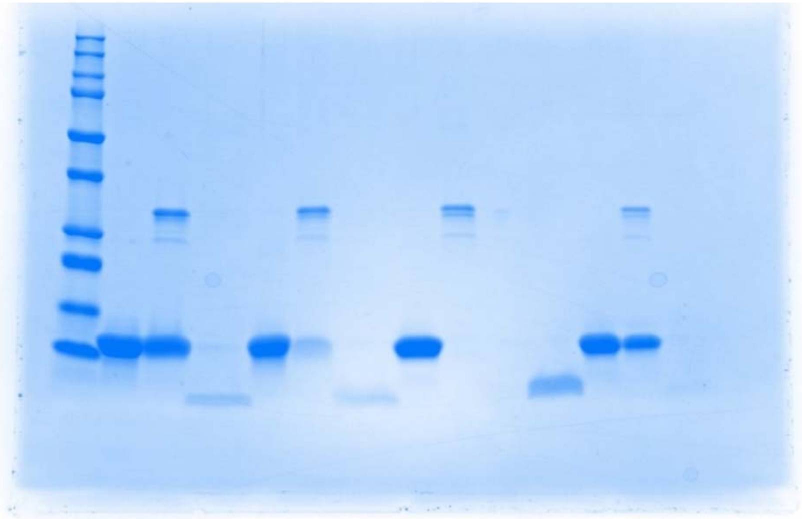

Figure 5S : Full-length gel of Fig. 5b.

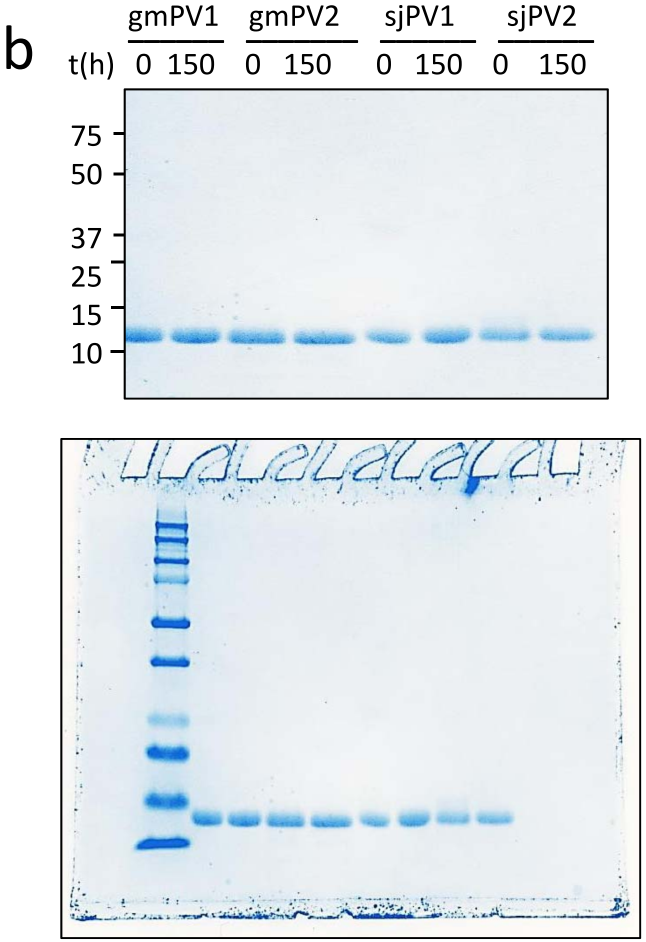

Figure 6S : Original membranes of Fig. 6b and 6c

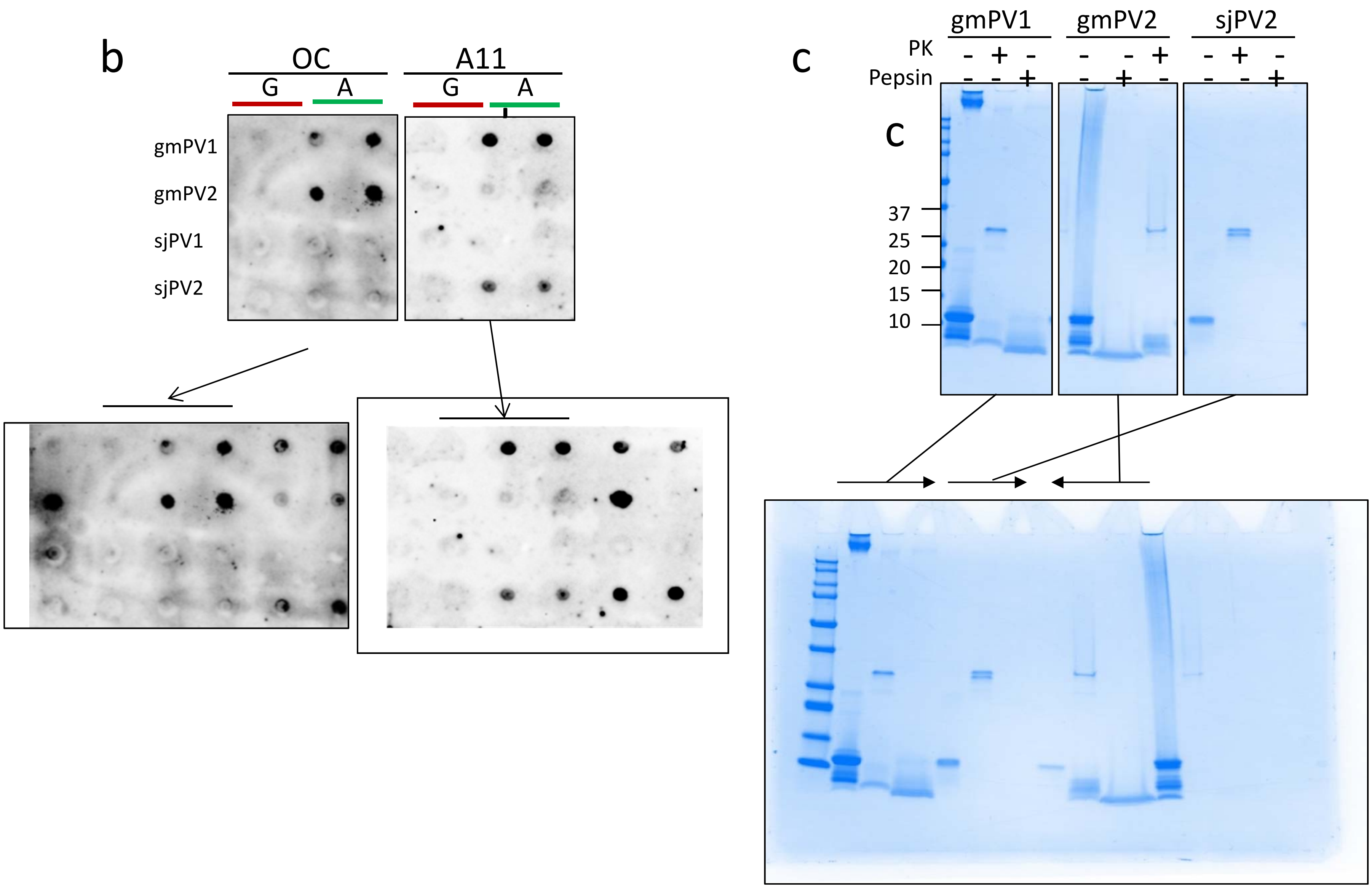

Figure 7S : Full size membranes of Fig. 7a, 7b and 7e.

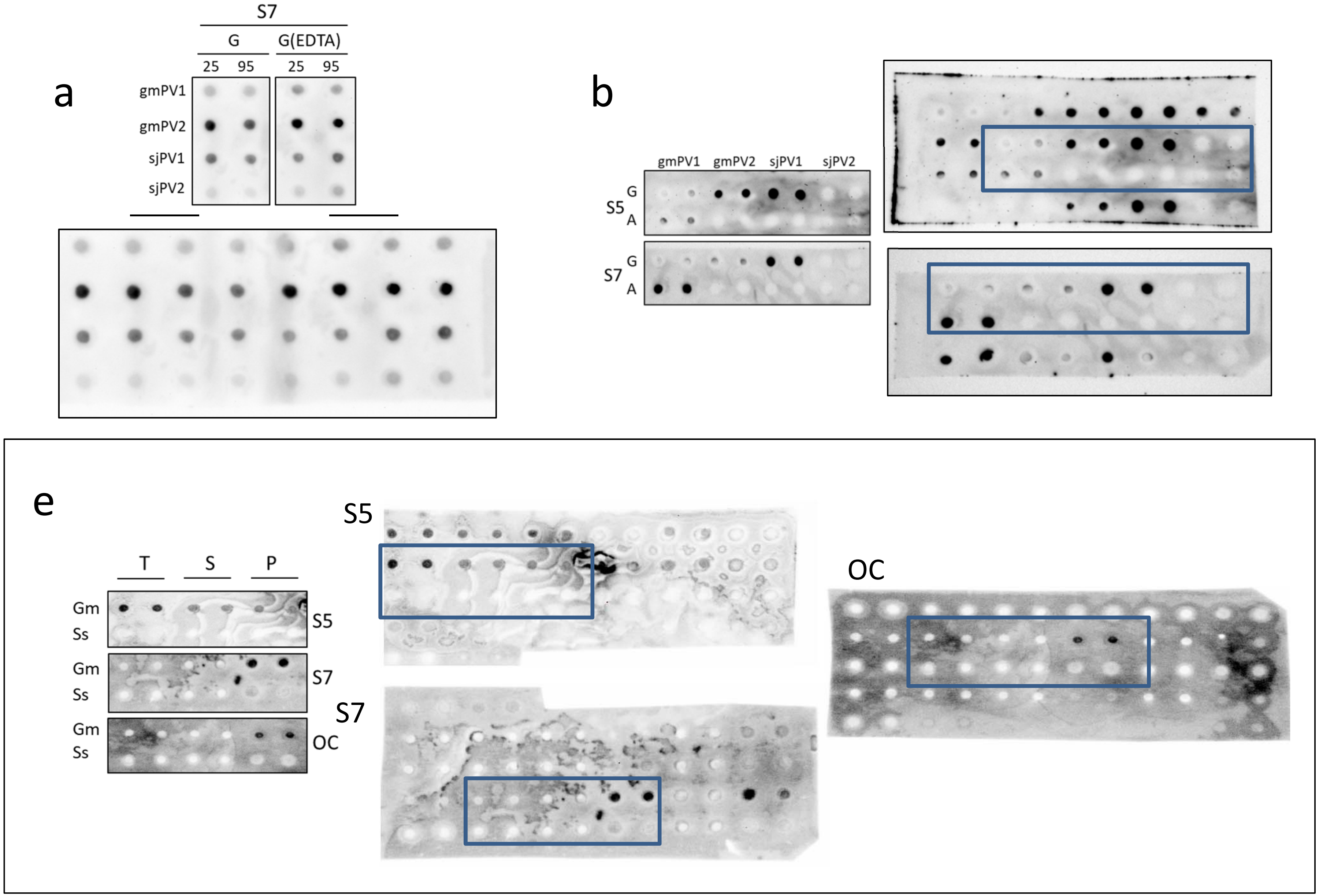

Supplement: Supplementary file 1 — Supplementary Material [file 41598_2019_52801_MOESM1_ESM.pdf]
